# *NDUFS7* variant in dogs with Leigh syndrome and its functional validation in a *Drosophila melanogaster* model

**DOI:** 10.1038/s41598-024-53314-7

**Published:** 2024-02-05

**Authors:** Matthias Christen, Anne Gregor, Rodrigo Gutierrez-Quintana, Jos Bongers, Angie Rupp, Jacques Penderis, G. Diane Shelton, Vidhya Jagannathan, Christiane Zweier, Tosso Leeb

**Affiliations:** 1 Institute of Genetics, Vetsuisse Faculty, University of Bern, Bern, Switzerland; 2 Department of Human Genetics, Inselspital, University of Bern, Bern, Switzerland; 3 Department for Biomedical Research (DBMR), University of Bern, Bern, Switzerland; 4 School of Biodiversity, One Health and Veterinary Medicine, College of Medical, Veterinary and Life Sciences, University of Glasgow, Glasgow, UK; 5 Vet-Extra Neurology, Stirling, UK; 6 Department of Pathology, School of Medicine, University of California San Diego, La Jolla, CA USA

**Keywords:** Genetics, Animal breeding, Neurological disorders

## Abstract

Two Jack-Russell Terrier × Chihuahua mixed-breed littermates with Leigh syndrome were investigated. The dogs presented with progressive ataxia, dystonia, and increased lactate levels. Brain MRI showed characteristic bilateral symmetrical T2 hyperintense lesions, histologically representing encephalomalacia. Muscle histopathology revealed accumulation of mitochondria. Whole genome sequencing identified a missense variant in a gene associated with human Leigh syndrome, *NDUFS7*:c.535G > A or p.(Val179Met). The genotypes at the variant co-segregated with the phenotype in the investigated litter as expected for a monogenic autosomal recessive mode of inheritance. We investigated the functional consequences of the missense variant in a *Drosophila melanogaster* model by expressing recombinant wildtype or mutant canine NDUFS7 in a ubiquitous knockdown model of the fly ortholog ND-20. Neither of the investigated overexpression lines completely rescued the lethality upon knockdown of the endogenous ND-20. However, a partial rescue was found upon overexpression of wildtype NDUFS7, where pupal lethality was moved to later developmental stages, which was not seen upon canine mutant overexpression, thus providing additional evidence for the pathogenicity of the identified variant. Our results show the potential of the fruit fly as a model for canine disease allele validation and establish *NDUFS7*:p.(Val179Met) as causative variant for the investigated canine Leigh syndrome*.*

## Introduction

Subacute necrotizing encephalopathy, or Leigh syndrome, was first described in human patients in 1951^1^. This predominantly neurological disease encompasses a broad spectrum of mitochondrial encephalopathies, such as defects in oxidative phosphorylation complexes, thiamine transport, or disorders of pyruvate metabolism^2^. A total of 113 gene-disease relationships have been established for the Leigh syndrome spectrum, 16 of which are linked to the mitochondrial DNA^3^. The first clinical signs are most frequently observed in children after a metabolic challenge, such as an acute infection^4^. Patients show developmental regression, dystonia, ataxia, and dysphagia. Clinical progression is then often rapid, and death may occur already in infancy^4^. One of the main criteria for early diagnosis of the disease are bilateral and symmetrical T2 hyperintense lesions involving basal ganglia, thalamus, midbrain, and brainstem on magnetic resonance imaging (MRI)^2^, corresponding to encephalomalacia. These lesions combined with evidence of mitochondrial dysfunction, such as elevated lactate levels, raise the suspicion of Leigh syndrome, which then can be confirmed by genetic testing.

In dogs, only two genetic variants have been associated with Leigh syndrome thus far (OMIA #001097-9615)^5^. In Alaskan Huskies, an *SLC19A3*:c.624delinsTGCAA variant leads to multifocal CNS disease with seizures, neurological deficits, blindness, and dysphagia^6^. The same gene is affected in Yorkshire terriers with juvenile-onset necrotizing encephalopathy, for which a homozygous c.205_210delins35 variant was identified in affected dogs^7^.

In this study, two Jack-Russell Terrier × Chihuahua (Jack-Chi) littermates were presented with clinical and magnetic resonance imaging (MRI) signs strongly resembling human disease of the Leigh syndrome spectrum. Our goal was to characterize the clinical, radiological, and histopathological phenotype, and to investigate a possible underlying causative genetic defect. The identified *NDUFS7* candidate variant was then further evaluated through overexpression of wildtype or mutant canine NDUFS7 in a previously described UAS-Gal4 mediated fruit fly ND-20-knockdown model^8^.

## Material and methods

### Ethics statement

All examinations and animal experiments were carried out after obtaining written informed owner’s consent and in accordance with local laws, regulations, and ethical guidelines. Dog blood samples were collected with the approval of the Cantonal Committee for Animal Experiments (Canton of Bern, Switzerland; permit BE 71/19).

### Animal selection for genetic analysis

This study was conducted with a total of 93 canine DNA samples, comprising the two affected dogs under investigation and their parents, all of which were Jack-Russell Terrier × Chihuahua (Jack-Chi) mixed breed dogs. Furthermore, samples of 39 purebred Chihuahuas and 50 purebred Jack Russell Terriers without reports of similar neurological phenotypes were retrieved from the Vetsuisse Biobank and used as unaffected controls.

### Clinical investigations

Two Jack-Chi littermates originating in the United Kingdom were investigated. Case 1 was male entire and case 2 was female entire. Both dogs presented at 4 months of age. Both parents and the two remaining littermates (one male and one female) were reportedly healthy. Case 1 was presented to the Small Animal Hospital of the University of Glasgow (Glasgow, UK) and case 2 to Vet-Extra Neurology (Stirling, UK). Blood was taken for hematology and serum biochemistry. A cerebrospinal fluid sample was taken for total and differential cell counts and protein levels. In case 1, blood and CSF lactate levels were additionally measured. Finally, for both cases urine was submitted for organic acid analysis to an external human laboratory.

### Magnetic resonance imaging

MRI of the brain was performed in both cases with 1.5 Tesla machines (1.5 T Magnetom, Siemens, Erlangen, Germany and 1.5 T Gyroscan ACS NT, Philips Medical System, Eindhoven, The Netherlands). Sequences included T2-weighted sagittal, dorsal and transverse views, and the following transverse views: fluid attenuated inversion recovery (FLAIR), Gradient echo (t2*), diffusion weighted imaging (DWI), T1-weighted pre- and post-contrast (gadopentate dimeglumine; Magnevist, Bayer Schering Pharma AG, Berlin, Germany).

### Necropsy and histopathology

Case 1 was submitted for necropsy examination. After macroscopic examination, representative samples were collected for routine histopathological examination of hematoxylin and eosin (H&E) stained sections. Additionally, skeletal muscles (biceps femoris and gastrocnemius) were precooled in isopentane followed by freezing in liquid nitrogen. Cryosections were evaluated using a standard panel of histochemical stains and reactions including the mitochondrial specific reactions succinic dehydrogenase and cytochrome C oxidase.

### DNA extraction and whole-genome resequencing

Genomic DNA was extracted from EDTA blood using the Maxwell RSC Whole Blood DNA kit in combination with the Maxwell RSC instrument (Promega, Dübendorf, Switzerland). An Illumina TruSeq PCR-free library with ~ 420 bp insert size was prepared from case 1. We collected 278 million 2 × 150 bp paired end reads on a NovaSeq 6000 instrument (31.7 × coverage). Mapping to the UU_Cfam_GSD_1.0 reference genome assembly was performed as described^9^. The sequence data were deposited under study accession PRJEB16012 and sample accession SAMEA10833663 at the European Nucleotide Archive.

### Variant calling and filtering, in silico functional predictions

Variant calling was performed using the GATK HaplotypeCaller^10^ in gVCF mode as described^9^. To filter for private variants in the affected dogs, we used publicly available genome sequences from 1479 control dogs of diverse breeds^9,11^ (Table S1). Sequences derived from Chihuahuas or Jack Russell Terriers were excluded as controls for variant filtering. Predictions of functional effects of the called variants were obtained with SnpEff software^12^ together with the UU_Cfam_GSD_1.0 reference genome assembly and NCBI annotation release 106.

PredictSNP^13^, PROVEAN^14^, and MutPred2^15^ were used to predict biological consequences of the candidate protein variant.

### PCR and Sanger sequencing

The candidate variant, *NDUFS7*:XM_038568001.1:c.535G > A, was genotyped by direct Sanger sequencing of PCR amplicons. A 340 bp PCR product was amplified using AmpliTaqGold360MasterMix (Thermo Fisher Scientific, Waltham, MA, USA) with the addition of 10% 360 GC Enhancer (Thermo Fisher Scientific) to the reaction volume, and primers Dog_NDUFS7_F and R (Table S2). Ethanol precipitation was used to purify the sequencing reactions, and an ABI 3730 DNA Analyzer (Thermo Fisher Scientific) was used for analysis of Sanger sequences. The raw sequence data were analyzed with the Sequencher 5.1 software (GeneCodes, Ann Arbor, MI, USA).

### Fly lines

Flies were kept on standard food made of agar, cornmeal, sugar, and yeast. All fly work was carried out at room temperature (23 °C). Knockdown and overexpression were achieved utilizing the UAS/GAL4 system^16^.

We established two UAS transgenic lines, one each for overexpression of wildtype canine NDUFS7 and the mutant ^179^Met allele, termed dog-wt and dog-mut, respectively. Expression constructs were generated by RT-PCR amplification of dog muscle cDNA. Primers that were used for the amplification and for mutagenesis are given in Table S2. Products were cloned into a pUASTattB fly expression vector (FlyORF^17^). After sequence verification, plasmids were used for generation of transgenic flies at FlyORF Zurich^18^. The respective control line was obtained from the Bloomington Drosophila Stock Center (24749 (Con-OE)). The RNAi knockdown line for ND-20 (101881/KK, KD ND-20) and the respective control (60100, Con-KD) were obtained from the Vienna Drosophila Resource Center (VDRC). The Actin-Gal4/Tm3b Sb Tb driver line for ubiquitous knockdown was assembled in house.

Double transgenic fly lines (KD ND-20 combined with dog-wt or dog-mut) were generated using a double balancer line (Kr/CyO;D/Tm6c) and termed KD-OE-WT and KD-OE-MUT (Figure S1 and Table S3).

A mating scheme was carried out to obtain flies with the ND-20 knockdown on chromosome 2 and the NDUFS7 overexpression constructs on chromosome 3 as shown in Figure S1.

#### Eclosion rate and pupae development

For analysis of eclosion rate and pupal development, ubiquitous dosage manipulation with the Actin-Gal4 / Tm3 Sb Tb driver was induced in the KD ND-20, dog-wt, dog-mut, KD-OE-WT and KD-OE-MUT flies, and their respective control lines. Crosses were carried out at 23 °C. Beginning at 12 days after mating when the flies started hatching, the number of hatched flies was counted every 48 h, until no more offspring hatched. Counted flies were removed from the vials. After the last count, the total number of empty pupae (flies hatched) and non-empty pupae was additionally counted. Non-empty pupae were distinguished between light (early pupal developmental stage) and dark puparium (late pupal developmental stage) to determine their developmental stage. All crosses with the driver line were repeated in an independent experiment. Statistical analysis was done with Chi-square tests in Excel spreadsheets to determine statistical significance between developmental stages of different strains.

## Results

### Clinical and neurological phenotype characterization

Both puppies presented with a 1-week history of progressive, generalized cerebellar and proprioceptive ataxia (Video S1). Case 1 also showed episodic dystonia with periods of sustained contraction of the left pelvic limb, and owners of case 2 reported recent concerns about poor vision and changes in house training. Physical examination was unremarkable in both cases. Neurological examination was very similar in both cases with normal mentation and cranial nerve function, except for a bilateral absence of the menace response. Cerebellar ataxia and intention tremors were evident. Postural reactions were decreased in all limbs. Segmental spinal reflexes were normal. No significant pain or discomfort was elicited during palpation of the vertebral column. The neuro-anatomical localization was multifocal with cerebellar, forebrain and possibly brainstem involvement.

Hematology and serum biochemistry (including ammonia) were within normal ranges. A CSF sample from the cerebromedullar cistern of case 1 revealed a normal cell count, yet an albuminocytological dissociation (protein levels: 59.4 mg/l; reference range < 25 mg/l). Furthermore, serial blood lactate measurements in case 1 consistently showed increased levels ranging from 6.8 to 9.5 nmol/l (normal < 2.5 nmol/l). Lactate levels in the CSF were also increased (12.6 nmol/l, reference < 2.5 nmol/l). Finally, both cases exhibited moderate lactic aciduria upon analysis of the urine organic acids.

**Figure 1:**
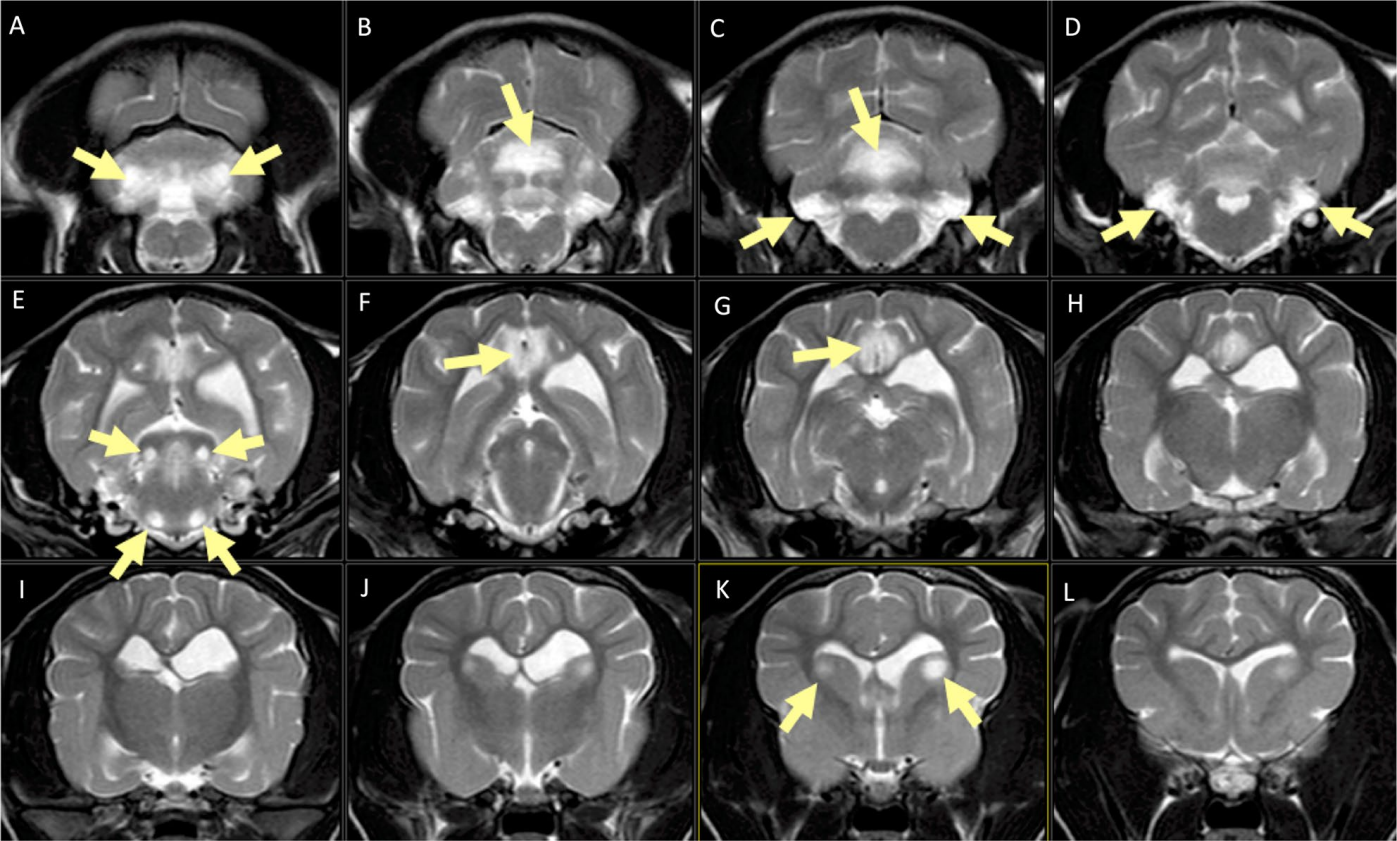
Transverse T2-weighted MRI images of case 2 from caudal to rostral, showing the bilateral and symmetrical T2-weighted hyperintense lesions. (**A**–**D**) Cerebellar cortex, white matter and cerebellar nuclei (arrows). (**E**) Rostral colliculi and brainstem nuclei (arrows). (**F**–**H**) Cingulate gyri (arrows). (**J**–**L**) Caudate nuclei.

**Figure 2:**
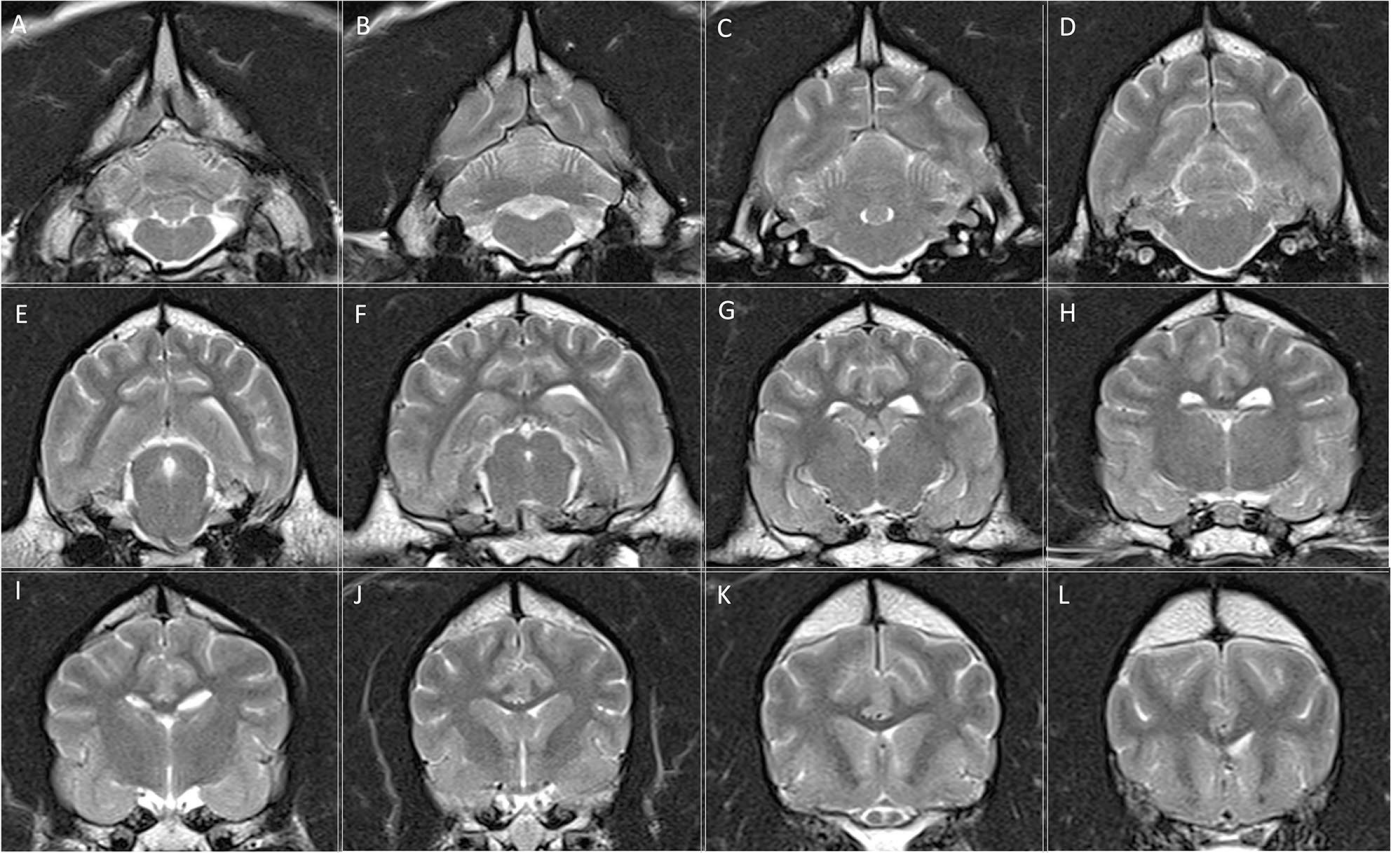
Transverse T2-weighted MRI images of a normal control dog at the same levels as in Fig. 1 for comparison.

MRI of the brain showed very similar findings in both cases. There were well-defined, rounded, multifocal, and bilaterally symmetrical intra-axial lesions, which were hyperintense on T2-weighted and FLAIR, isointense on T1-weighted images, showed diffusion restriction on diffusion weighted imaging, and very mild enhancement in some lesions in T1-weighted images after contrast (Figs. 1 and 2). The lesions affected the caudate nuclei, cingulate gyri, rostral and caudal colliculi, lateral lemniscus, cerebellar cortex, cerebellar white matter, cerebellar nuclei, and multiple brainstem nuclei (olivary nuclei and pontine nuclei).

Case 1 received vitamin B1 and B12 injections, but unfortunately, both cases showed progressive deterioration with progression of clinical signs and the owners elected euthanasia soon after the investigations. Case 1 was submitted for necropsy.

### Histopathology of case 1

**Figure 3:**
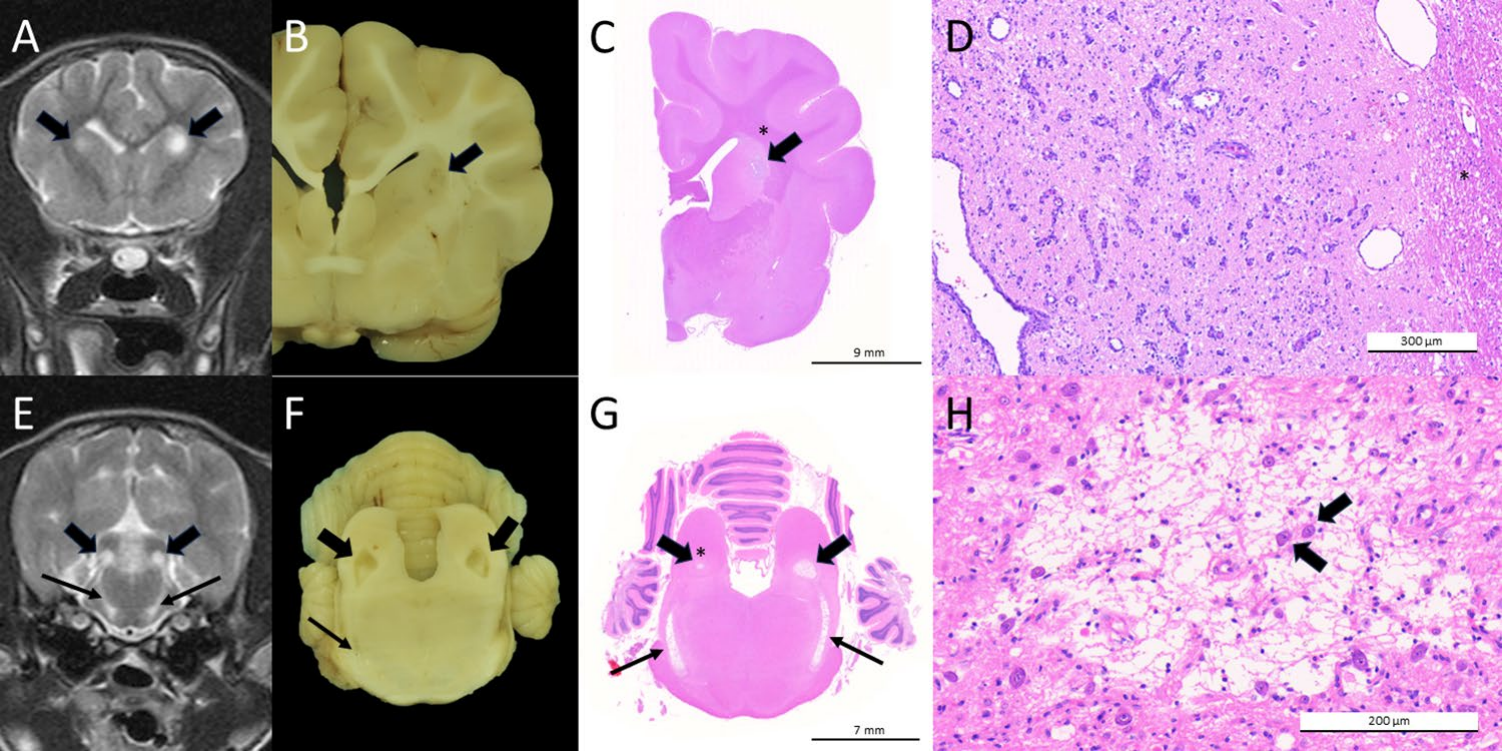
Brain, corresponding imaging (transverse T2-weighted MRI images; **A**,**E**), gross pathology (fixed brain; **B**,**F**) and histopathology (H&E stain; **C**,**D** and **G**,**H**) of case 1. (**A**–**D**) lesions in caudate nucleus (arrows), (**E**–**H**) lesions in caudal colliculi (arrows) and lateral lemnisci (thin arrows), with D close-up of lesion in caudate nucleus (asterisk indicates internal capsule in **C** and **D**) and H close-up of lesion in left caudal colliculus (asterisk in **G**). The lesion in the caudate nucleus, difficult to appreciate on gross examination (**B**), is characterized by vascular proliferation (**D**), whilst those in the caudal colliculi and lateral lemnisci exhibit a substantial loss of the neuropil and cavitation, with sparing of individual intralesional neurons (**H**, arrows).

Histopathological investigations revealed the lesions on MRI to correspond to bilaterally symmetrical regions of subacute to chronic necrosis characterized by pronounced vascular proliferation combined with mild rarefaction of the neuropil, Gitter cell infiltrates and predominantly peripheral gliosis, progressing to substantial loss of the neuropil, with cavitating lesions occasionally also exhibiting spared intralesional neurons (Fig. 3).

**Figure 4:**
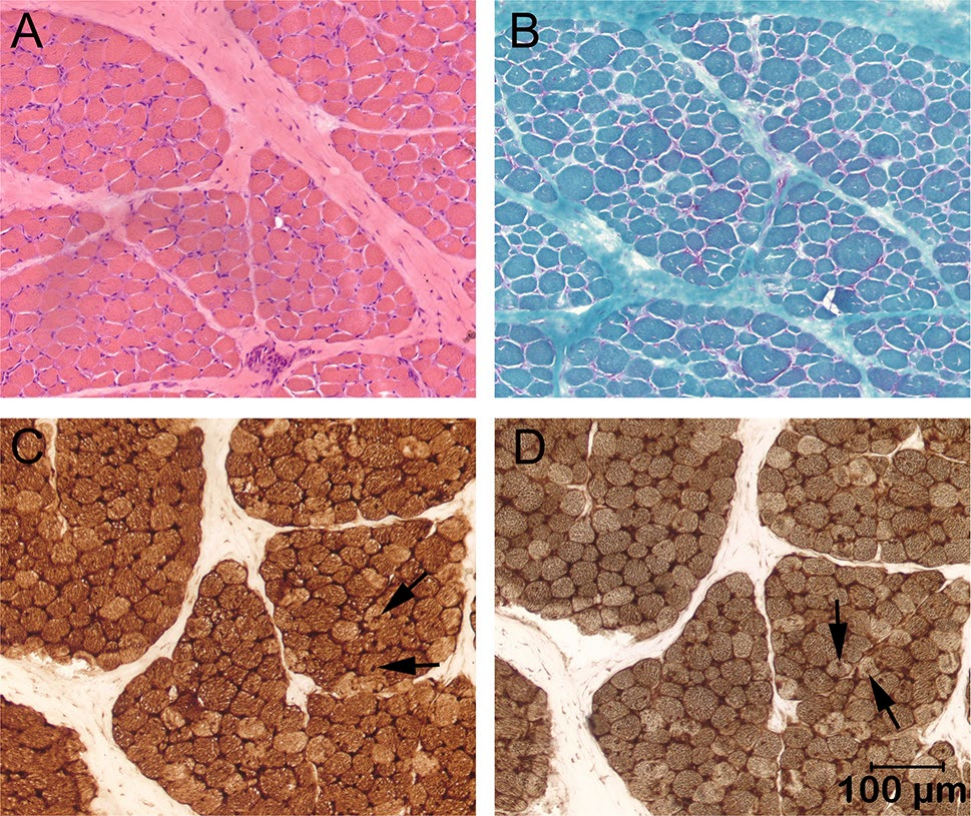
Cryosections from the biceps femoris muscle of case 1. (**A**) H&E, (**B**) modified Gomori trichrome, (**C**). mitochondrial specific reactions cytochrome C oxidase (COX), and (**D**) combined cytochrome C oxidase and succinic dehydrogenase (SDH). Variability in myofiber size was present in A and B without obvious ragged red fibers. Punctate dark brown intramyofiber deposits highlighted by the COX and combined COX and SDH reactions indicating mitochondrial accumulations are indicated with arrows.

Representative cryosections from the biceps femoris muscle showed no specific abnormalities with H&E, as well as with modified trichrome stains, except for variability in myofiber size (Fig. 4A,B). Ragged red fibers were not obvious. Using the mitochondrial specific reactions cytochrome C oxidase and combined cytochrome C oxidase and succinic dehydrogenase reactions, several punctate areas of dark brown positive deposits consistent with mitochondrial accumulations were noted (Fig. 4C,D).

### Genetic analysis

As two dogs from a single litter with healthy parents were affected by the same phenotype, we hypothesized a monogenic autosomal recessive mode of inheritance. The genome of case 1 was sequenced and searched for homozygous private protein changing variants by comparing the variants to 1479 control genomes (TableS 1, S4). Further prioritization of resulting variants was then done based on known candidate genes for Leigh syndrome in humans^2^.

**Table 1:** Results of variant filtering in an affected dog against 1479 control genomes.

Filtering step	Homozygous variants
All variants in the affected dog	2,702,622
Private variants	1842
Protein-changing private variants	21
In functional candidate genes for Leigh syndrome in humans	1

**Figure 5:**
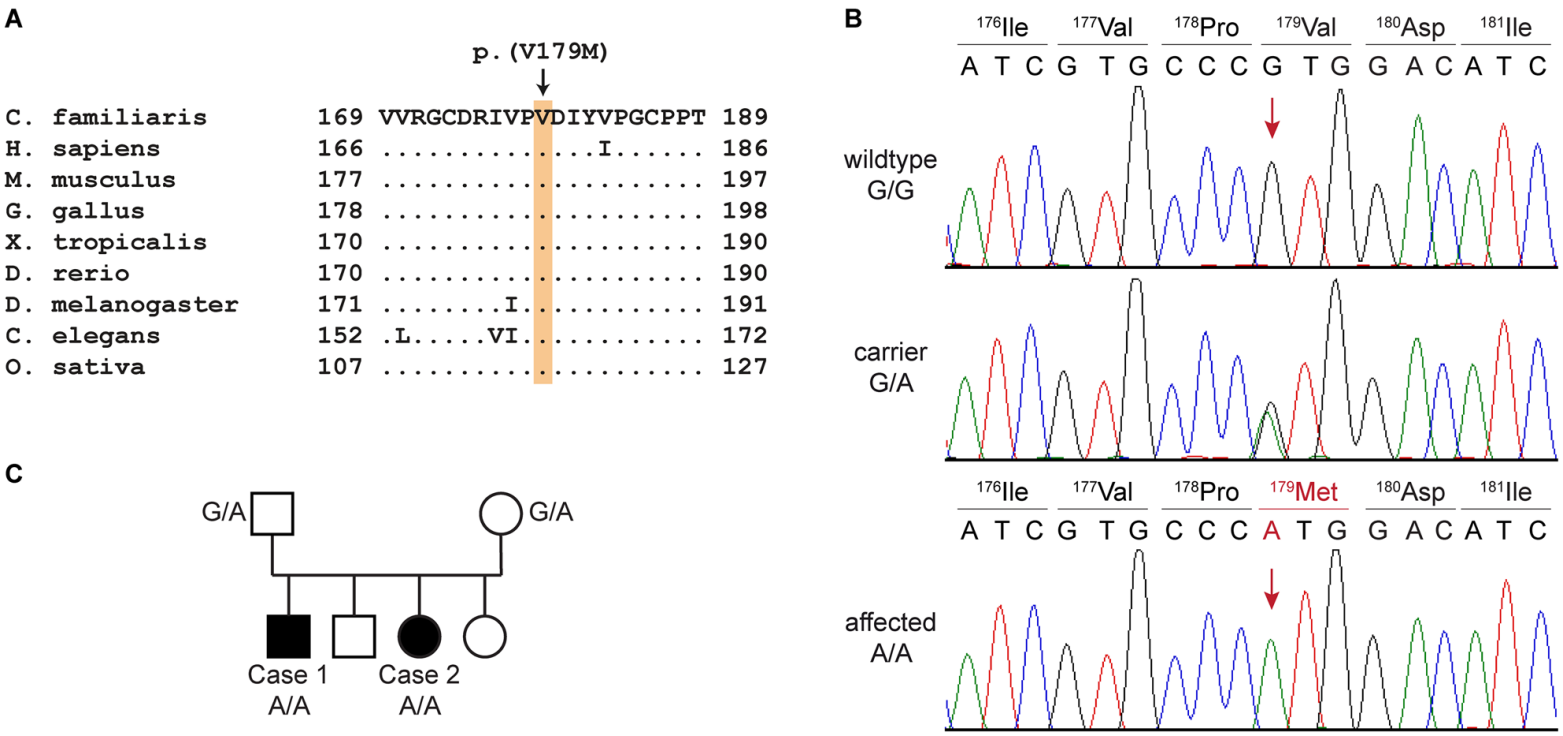
Details of the *NDUFS7*:c.535G > A variant (p.Val179Met). (**A**) Multiple-species alignment of the *NDUFS7* amino acid sequences near the V179M variant. The variant affects a highly conserved valine residue. Accession numbers: dog (*Canis familiaris*) XP_038423929.1; human (*Homo sapiens*) NP_077718.3; mouse (*Mus musculus*) NP_083548.1; chicken (*Gallus gallus*) XP_001234054.3; frog (*Xenopus tropicalis*) NP_001072396.1; zebrafish (*Danio rerio*) NP_001186841.1; fruit fly (*Drosophila melanogaster*) NP_727921.1; roundworm (*Caenorhabditis elegans*) NP_492445.1; Japanese rice (*Oryza sativa Japonica Group*) NP_001056143.2. (**B**) Representative electropherograms of a wildtype dog, a heterozygous carrier, and an affected dog. The amino acid translations of the wildtype and mutant alleles are indicated. (**C**) Pedigree of the Jack-Chi dogs with Leigh syndrome. Affected dogs are indicated with filled symbols. Genotypes at the *NDUFS7*:c.535G > A variant are indicated for all dogs, from which a DNA sample was available.

The bioinformatic analysis identified 21 homozygous private protein-changing variants in the affected dog. Only one of them was located in a functional candidate gene for Leigh syndrome, in *NDUFS7*. This variant, Chr20:57,913,322G > A or XM_038568001.1:c.535G > A, is predicted to result in an amino acid substitution in a highly conserved region of the encoded NADH:ubiquinone oxidoreductase core subunit S7, XP_038423929.1:(p.Val179Met) (Fig. 5). The other 20 private protein-changing variants were not located in genes known to cause phenotypes resembling mitochondrial encephalopathies or Leigh syndrome in humans, mice, or domestic animals (Table S4).

The valine-to-methionine substitution was predicted to be pathogenic and deleterious by several in silico prediction tools (PredictSNP probability for pathogenicity: 87%; MutPred2 score: 0.770 with several predicted altered molecular mechanisms; PROVEAN score: − 2.995).

The variant was genotyped in the family (Fig. 5C) and an additional cohort of 39 Chihuahuas and 50 Jack Russell Terriers. Both cases were homozygous for the mutant allele, while both parents were heterozygous, as would be expected for a monogenic autosomal recessive mode of inheritance. None of the 89 additionally genotyped unrelated, healthy control dogs carried the mutant allele.

### Pupal development and eclosion rate

We evaluated the effect of the identified candidate variant with a rescue experiment in a fly model. The canine *NDUFS7*:XP_038423929.1:p.(Val179Met) missense variant is located in a highly conserved region with respect to its orthologous ND-20 protein in *Drosophila melanogaster*. Ubiquitous *ND-20* knockdown leads to near complete lethality^8^. Neither simultaneous overexpression of wildtype nor mutant canine NDUFS7 protein was able to rescue lethality due to knockdown of ND-20. No adult flies or empty pupae were found in any of the vials with any combination of ND-20 knockdown and overexpression of canine NDUFS7 upon simultaneous ubiquitous dosage manipulation. In comparison, the total eclosion rates in the Con-KD and Con-OE control lines were not impaired with 88.2% and 93.2%, respectively, and ubiquitous overexpression of NDUFS7 with dog-wt or dog-mut alone did not affect survival.

**Figure 6:**
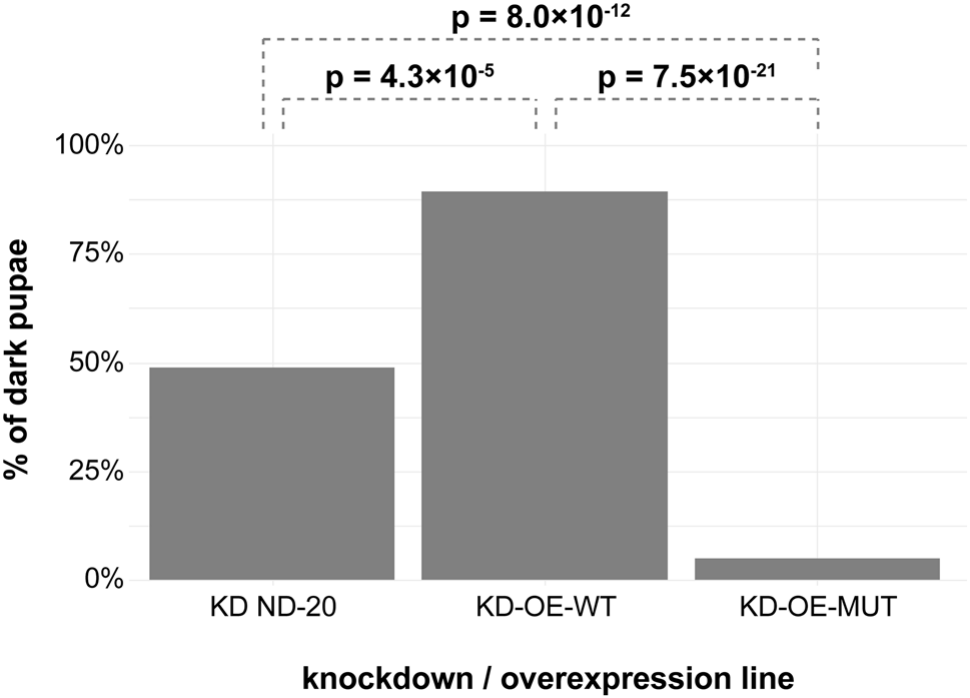
Influence of wildtype or mutant transgene overexpression in ND-20 knockdown lines. Percentages of dark pupae in all unhatched pupae are shown. Chi-square tests were used to determine statistical differences between different lines. KD-OE-MUT showed impaired development compared to the KD ND-20, while the KD-OE-WT line showed improved development, but still complete lethality before eclosion upon ubiquitous dosage manipulation. The numerical values for counted pupae and flies are provided in Table S5.

Hence, the stages of developmental arrest upon manipulation with the different lines were analyzed. We evaluated the proportion of the developmentally more advanced dark pupae compared to the total number of non-empty pupae upon simultaneous, ubiquitous knockdown of ND-20 and overexpression of either WT or mutant canine NDUFS7 (KD-OE-WT and KD-OE-MUT) (Fig. 6). The KD-OE-WT condition showed a partial rescue with more advanced pupal development when compared to the knockdown of ND-20 alone. The KD-OE-MUT condition not only failed to show this partial rescue, but pupae were almost exclusively light, showing an even earlier arrest of development than upon ND-20 knockdown alone. These differences were consistently observed in two independent sets of experiments.

## Discussion

In the course of this study, the *NDUFS7*:p.(Val179Met) missense variant was identified in two Jack-Chi puppies with Leigh syndrome. The *NDUFS7* gene encodes the NADH:ubiquinone oxidoreductase core subunit S7 of the mitochondrial respiratory complex I in the oxidative phosphorylation system^19^. Complex I is the largest electron transfer chain enzyme and is the main entry point of electrons into the respiratory chain^20^. It is made up of 44 different proteins, and variants in at least 39 genes have been described to cause complex I deficiency with Leigh syndrome as frequent outcome of the encountered clinical spectrum (OMIM PS252010)^20^. Several human *NDUFS7* variants specifically cause ‘mitochondrial complex I deficiency, nuclear type 3’ (OMIM #618,224)^21–23^. The known pathogenic variants were speculated to affect the electron transfer from NADH to ubiquinone either through impaired catalytic activity or defective assembly of complex I^21,23,24^. The clinical consequences for the affected patients were initial respiratory signs followed by muscular weakness and hypotonia, elevated lactate levels, and characteristic, T2-hyperintense MRI lesions consistent with Leigh syndrome^21–23,25^.

In dogs, clinical phenotypes sharing similarities with Leigh syndrome have been reported in American Staffordshire bull terriers^26,27^, Alaskan huskies^6^, Yorkshire terriers^7^, Australian cattle dog^28^, a Shih Tzu^29^ and a mixed-breed dog^30^. Canine cases tend to present at an early age with most reported cases being younger than 1.5 years of age^6,7,26–30^. Similar to human patients, affected dogs show bilateral symmetrical lesions affecting the gray matter in specific areas of the brain and spinal cord, but the exact distribution of the lesions within the neuraxis varies between the different breeds. For example, the thalamus is commonly affected in Alaskan huskies and Yorkshire terriers with variants in *SLC19A3* and was not affected in the dogs from this report^6,7^. Interestingly, bilateral symmetrical changes affecting the cerebellar nuclei were reported in Bullmastiff dogs with a variant in the *MFF* gene encoding mitochondrial fission factor^31^. Lactate was significantly increased in blood and CSF in one of the cases from this study and is a very common finding in humans with Leigh syndrome, reported in around 70% of the cases^32^. Increased lactate levels were also reported in the American Staffordshire bull terriers^26,27^ with severe necrotizing encephalopathy, in Border terriers^33^ that presented with a spongiform leukoencephalopathy with bilateral symmetrical changes in MRI and in which a mitochondrial disorder was also suspected, and interestingly in a young Jack Russell terrier^34^ with mitochondrial myopathy, but in which no investigation of the central nervous system was performed.

Further, in muscle cryosections in the Jack Russell terrier with mitochondrial myopathy, ragged-red fibers were noted with the modified Gomori trichrome stain^34^. Such fibers were not found in muscle cryosections from the mixed breed dog of this report. However, punctate COX and SDH positive intramyofiber deposits consistent with mitochondrial accumulations were observed. Diagnosis of a complex I deficiency can be achieved by measuring enzymatic activity using spectrophotometric assays in patient-derived cells and tissues. Unfortunately, it was not possible to perform this test with the samples obtained from the dogs reported in this study^35^.

The canine Val179Met change was predicted to be pathogenic or deleterious by all used in silico prediction tools. To gain more insight into the role of the Val179Met missense variant in the pathogenesis of Leigh syndrome, we chose the fruit fly as a model organism. Given that a previously investigated ND-20/NDUFS7 knockdown in *Drosophila melanogaster* results in near complete lethality before eclosion^8^, we wanted to test if overexpression of wildtype or mutant canine NDUFS7 protein might be able to rescue this phenotype. This is an established approach to investigate pathogenic potential of missense variants in human conditions^36,37^. Neither the pure knockdown condition of ND-20, nor overexpression of the two tested NDUFS7 rescue lines KD-OE-WT and KD-OE-MUT were able to produce viable flies. This might indicate that either the overexpression of the transgene together with the knockdown of ND-20 was not strong enough, or that the knockdown phenotype might already have been too strong at the chosen temperature of 23 °C to produce sufficient functional protein in this experiment. As Floriel et al. already pointed out, it might be challenging to obtain full rescue in a developmentally lethal phenotype of this severity^8^. Nonetheless, clear differences in the progression of pupal development before mortality were seen upon simultaneous overexpression of canine NDUFS7 (KD-OE-WT), when compared to ND-20 knockdown alone (Fig. 6). Interestingly, simultaneous overexpression of mutant canine NDUFS7 (KD-OE-MUT) was not able to improve pupal development, indicating loss of function of the overexpressed mutant allele. The fact that pupal development in this combined condition was actually more severely impaired than upon pure knockdown of ND-20 might even hint to a dominant negative effect of the canine mutant, at least in the investigated *Drosophila* context with a residual level of ND-20. The fully recessive mode of inheritance in dogs suggests that in dogs a pure loss-of-function effect is more likely than a dominant negative effect.

Taken together, the clinical, imaging and histopathological findings in combination with the genetic data and the results of our functional experiments in *Drosophila melanogaster* establish the *NDUFS7*:c.535G > A, p.(Val179Met) missense variant as disease causing in the two investigated Jack-Chi puppies. To the best of our knowledge, the affected dogs represent the first domestic animals described with a naturally occurring genetic variant causing complex I deficiency. The identification of a causative variant enables genetic testing and the detection of heterozygous carriers. Thus, unintentional breeding of further affected dogs can be prevented. If similarly affected dogs should appear in the Jack-Chi population or possibly even purebred Chihuahuas or Jack Russell Terriers, this genetic test can also be used to quickly confirm the suspected diagnosis. Additionally, the studied dogs might serve as a spontaneous large animal model to further understand the pathogenesis of Leigh syndrome.

### Supplementary Information

Supplementary Table S1.

Supplementary Video S1.

Supplementary Table S4.

Supplementary Information.

## Data Availability

All data are contained in this manuscript and the supplementary files. Accession numbers for the whole genome sequence data are given in Table S1.
