# Supplementary material for: NDUFS7 variant in dogs with Leigh syndrome and its functional validation in a Drosophila melanogaster model
Source: Sci Rep. 2024 Feb 5;14:2975. doi: 10.1038/s41598-024-53314-7 (PMC10844639; doi:10.1038/s41598-024-53314-7)
Supplement: Supplementary file 4 — Supplementary Information. [file 41598_2024_53314_MOESM4_ESM.pdf]

NDUFS7 variant in dogs with Leigh syndrome and its functional validation in a *Drosophila melanogaster* model

Matthias Christen[1,a], Anne Gregor[2,3,a], Rodrigo Gutierrez-Quintana[4], Jos Bongers[4], Angie Rupp[4], Jacques Penderis[5], G. Diane Shelton[6], Vidhya Jagannathan[1], Christiane Zweier[2,3], Tosso Leeb[1,*]

1 Institute of Genetics, Vetsuisse Faculty, University of Bern, Bern, Switzerland

2 Department of Human Genetics, Inselspital, University of Bern, Bern, Switzerland

3 Department for Biomedical Research (DBMR), University of Bern, Bern, Switzerland

4 School of Biodiversity, One Health and Veterinary Medicine, College of Medical, Veterinary and Life Sciences, University of Glasgow, Glasgow, United Kingdom

5 Vet-Extra Neurology, Stirling, United Kingdom

6 Department of Pathology, School of Medicine, University of California San Diego, La Jolla, California, USA

Supporting Information

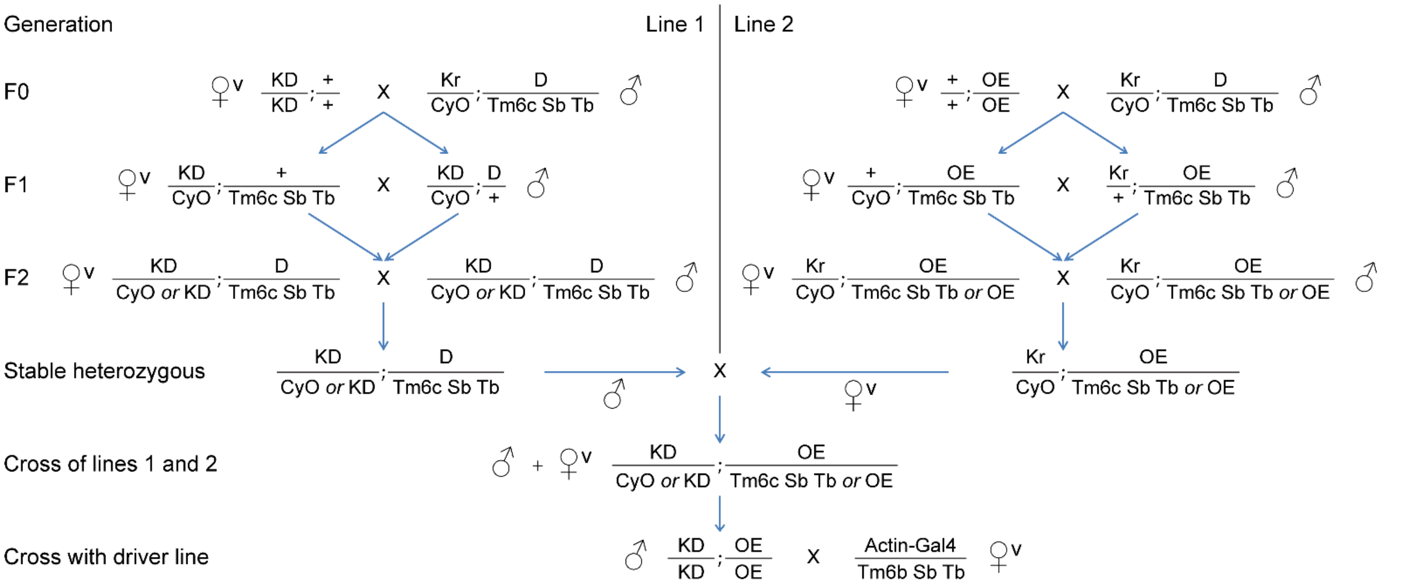

Figure S1. *Drosophila* mating scheme. Knockdown (KD) and overexpression (OE) lines are separately crossed into the double balancer line Kr/CyO; D/Tm6c Sb Tb to create stable double balanced lines. Lines for KD and OE (WT and Mut) are then combined into one line each. The ♀v indicates female virgins.

+: wild type
CyO: Balancer of chromosome 2 with CyO (Curly of Oster) marker = curled wings.
D: Dichaete = protruding wings
Kr: Krüppel = malformed eyes
Sb: Stubby = shortened back hair
Tb: Tubby = shorter and thicker body
Tm6c: Balancer of chromosome 3

Table S2. Primer sequences

Primer Name	Sequence	Use
Dog_NDUFS7_F	CCTGGCACCTGTTTGTACCT	Sanger sequencing of candidate variant
Dog_NDUFS7_R	CCTAAGACTTTGCCGTCGAG	Sanger sequencing of candidate variant
CFA_NDUFS7_F1	AGATCTGAGGTTGTTTGAAGGCCGAG	Amplification of dog muscle cDNA for UAS driven overexpression lines
CFA_NDUFS7_R1	GTGGGTGTATTGGGTTTATTGAC	Amplification of dog muscle cDNA for UAS driven overexpression lines
CFA_G535A_F	GACCGCATCGTGCCCATGGACATCTACGTG	Mutagenesis, Val179Met change in dog cDNA
CFA_G535A_R	CACGTAGATGTCCATGGGCACGATGCGGTC	Mutagenesis, Val179Met change in dog cDNA
cfa_NDUFS7_qPCR_1F	AGTCCTCTGCACTTCCCAG	qPCR for control of overexpression
cfa_NDUFS7_qPCR_1R	CATCATCTCCACGGCACAG	qPCR for control of overexpression

Table S3. Drosophila lines

Name	Line	Origin (Order number)
Actin-Gal4/Tm3b Sb Tb	Actin-Gal4/Tm3b Sb Tb	Assembled in house
Double balancer	Kr/CyO;D/Tm6c	Assembled in house
Con-KD	y,w[1118];P{attP,y[+],w[3`]	VDRC (60100)
KD ND-20	UAS-RNAi ND-20	VDRC (101881)
Con-OE	y[1] M{RFP[3xP3.PB] GFP[E.3xP3]=vas-int.Dm}ZH-2A w[*]; M{3xP3-RFP.attP}ZH-86Fb	Bloomington Stock Center (24749)
Dog-wt	wt;UAS-NDUFS7-WT (XP_038423929.1)	FlyORF (injection of Con-OE with pUAST-NDUFS7-WT construct)
Dog-mut	wt;UAS-NDUFS7-MUT (XP_038423929.1:(p.Val179Met))	FlyORF (injection of Con-OE with pUAST-NDUFS7-Mut construct)
KD-OE-WT	KD ND-20 + dog-wt	Assembled in house
KD-OE-MUT	KD ND-20 + dog-mut	Assembled in house

Table S5. Total count of flies and pupae

Fly count, 23 °C, both vials together								
**Females: ActinGal4/Tm3**	**Males →**	**KD ND-20**	**Dog-wt**	**Dog-mut**	**KD-OE-WT**	**KD-OE-MUT**	**Con-KD**	**Con-OE**
	Males sbtb	122	60	114	94	82	93	106
	Females sbtb	101	103	119	108	74	101	119
	Males wt	0	95	122	0	0	94	116
	Females wt	0	109	136	0	0	138	151

Pupae count, 23 °C, both vials together								
**Females: ActinGal4/Tm3**	**Males →**	**KD ND-20**	**Dog-wt**	**Dog-mut**	**KD-OE-WT**	**KD-OE-MUT**	**Con-KD**	**Con-OE**
	Empty, long	0	n.d.	n.d.	0	0	189	233
	Empty, short	222	n.d.	n.d.	180	160	186	228
	Dark, long	125	n.d.	n.d.	186	7	1	1
	Dark, short	7	n.d.	n.d.	5	1	4	3
	Light, long	131	n.d.	n.d.	22	139	2	16
	Light, short	9	n.d.	n.d.	5	3	3	19
